# Heavy metal removal from MSWI fly ash by electrokinetic remediation coupled with a permeable activated charcoal reactive barrier

**DOI:** 10.1038/srep15412

**Published:** 2015-10-21

**Authors:** Tao Huang, Dongwei Li, Liu Kexiang, Yuewei Zhang

**Affiliations:** 1 State Key Laboratory for coal mine disaster dynamics and control, Chongqing University, 400044, China; 2 College of Resource and Environmental Science, Chongqing University, Chongqing, 400044, China

## Abstract

This paper presents the investigations into the feasibility of the application of a remediation system that couples electrokinetic remediation (EKR) with the permeable reactive barrier (PRB) concept for municipal solid waste incineration (MSWI) fly ash with activated charcoal as the PRB material. The experimental results of this study showed that the proposed combined method can effectively improve the remediation efficiency and that the addition of the oxalic acid to the PRB media before the coupled system can further enhance the remediation process. In the optimization tests, the maximum removals of Zn, Pb, Cu and Cd were achieved under different experimental conditions. The voltage gradient and processing time were shown to have significant effects on the removal of Cu and Cd, whereas the addition of the oxalic acid had a more significant influence on the removal of Pb. Generally, the processing time is the most significant factor in changing the removal rates of HMs in the enhanced coupled system. In terms of the leaching toxicity, the specimen remediated by ENEKR + PRB showed the lowest leaching value for each HM in the S2 and S3 regions.

The gross capacity of municipal solid waste required for power generation in a power plant by burning increases exponentially every year in China. The significant need for electricity continues to increase, causing and accelerating the accumulation of fly ash produced by municipal solid waste incineration (MSWI)^1^,^2^. In many countries, the MSWI fly ash produced by the incineration of different raw sources is classified as hazardous waste due to its high content of heavy metals (HMs) and toxic chlorinated organics^3^,^4^,^5^. Currently, the traditional method of excavation and landfilling is still the primary method for disposing MSWI fly ash in China, which may require large amounts of municipal land and wide-spread contamination under certain conditions. Many techniques exist for the remedial treatment of heavy-metal-contaminated solids including physical separation and isolation, immobilization, flushing, toxicity reduction and phytoremediation. However, when considering these techniques for use with MSWI fly ash, problems including incomplete metal removal, high consumption of reagents and energy, low selectivity and the generation of secondary wastes are inevitable and are difficult to mitigate^6^,^7^,^8^.

In recent years, electrokinetic remediation has been shown to be an effective tool to clean up heavy-metal-contaminated fine-grained soils and has attracted significant attention for heavy metal removal in low-permeability soils^9^,^10^. Electrokinetic remediation can be applied *in situ* or *ex situ* for contaminated soils, sediments, sewage sludge and mine tailing and typically involves passing a low direct electrical current through the matrix by imbedding electrode pairs in the contaminated solid. The removal of contaminants from porous solutions by EKR is primarily accomplished by electrolysis, electromigration, electroosmosis and electrophoresis^11^,^12^. The solid type, contaminant type and concentration, zeta potential, electrode spacing and coupled enhancement techniques are considered to be the primary factors that affect the removal efficiencies of organic and inorganic contaminants from solid matrices. EKR technology is primarily used to remove and concentrate contaminants in the focusing area or in a reservoir solution; any subsequent poor handling procedures of the extracted contaminants can cause secondary or further contamination^9^,^13^. Thus, a necessity exists for EKR technology to be used in combination with other effective techniques^14^. Permeable reactive barriers (PRBs) developed in the early 1990 s and first used effectively in 1994 have gained popularity in the *in situ* treatment of groundwater contamination in recent years due to their excellent performance, low cost and simple control procedures^15^,^16^. Typically, a PRB system is placed across the flow path of a contaminated plume in a natural gradient to retain or degrade the contaminants in the plume flows by adsorption, precipitation and redox reactions between the pollutant and the barriers^17^. A variety of materials have been selected and used to prepare barriers, such as Fe(0), activated carbon, zeolite, goethite. In recent years, with the development of material research, carbonized food waste, lignocellulose from agricultural waste, composite materials, and bio-sorbents as developing and ecofriendly filling materials or adsorbents are also being applied in PRB processes^18^,^19^.

In recent years, combining electrokinetics and PRBs has been widely applied in the remediation of heavy metals and organic pollutants^20^,^21^. With the synergistic effects of this combined method, the force of the contaminant flow through the barrier is provided by electromigration, electroosmosis and electrophoresis, replacing the natural hydraulic gradient of groundwater. The pH gradient generated by the EKR process in the PRB may affect the chemical and electrochemical characteristics of the reactive media in the PRB^9^. Weng *et al.* investigated the effectiveness of a granular zero-valent iron (ZVI) PRB installed in the middle of a hyper-Cr^6+^-contaminated clay soil sample with electrochemical remediation. The amount of Cr^6+^ ions was shown to be significantly reduced by the ZVI PRB, converting them to Cr^3+^ ions^22^. Yuan and Chiang studied the removal mechanisms of arsenic from soil by EKR coupled with a PRB made of ZVI and FeOOH. The extraction efficiency for arsenic was increased by 60–120%, and the maximum efficiency was achieved with a FeOOH layer installed at the middle of the soil specimen^23^. Kimura *et al.* has remediated Cu-contaminated kaolinite by coupling EKR with a ferrite treatment zone (FTZ); 92% of the Cu ions in the contaminated kaolinite were found to migrate into the FTZ after 48 h of treatment^24^. Han *et al.* used EKR coupled with a PRB made of carbonized food waste (CFW) to detoxify Cu-contaminated kaolinite. Acetic acid was injected into the electrolyzer from the anode to elevate the removal efficiency during experimentation. The sorption capability of CFW used as the PRB reactive medium was found to be 4–8 times more efficient than that of zeolite^25^. Yuan *et al.* applied an enhanced EKR coupled with carbon nanotubes coated with cobalt (CNT-Co) for As^5+^ removal. Due to the higher sorption efficiency of As^5+^, which can be attributed to CNT-Co, a 62% remediation efficiency was achieved after the experiment^26^.

Although combining EKR and PRBs for the disposal of contaminated soil has become commonly used, reference to the remediation of MSWI fly ash with this method was scarce. To increase the number of applications of this coupled technique and investigate the feasibility of remediating toxic fine-grain MSWI fly ash, EKR technology coupled with an activated-charcoal PRB was proposed in this study to enhance the removal efficiency of MSWI fly ash. Batch tests of aqueous equilibrium adsorption under different conditions of pH, adsorbent dosage and processing time before the EKR-PRB process was conducted to understand the removal behavior of HMs by the PRB media and to obtain the proper experimental parameters for the subsequent coupled tests. The trends of the pH and the electric density from the EKR and EKR-PRB tests were reported and analyzed, respectively, to study the migration characteristics of HMs in the different processing electrolyzers. Then, the removal efficiencies and leaching toxicities of the HMs in the fly ash matrices after the experiments were evaluated to determine the removal of the HMs and to directly measure the remediation effect of the coupled system in the samples. X-ray diffraction, scanning electron microscopy with an energy dispersive spectrometer and FTIR spectroscopy were used to further analyze and understand the mechanisms of heavy metal removal from MSWI fly ash particles via EKR coupled with activated-charcoal PRBs.

## Materials and Methods

### Preparation and characteristic analysis of fly ash and PRB media

The MSWI fly ash samples were collected from the TongXing Waste Power Generation Plant in TongGuXi town, Beibei county, Chongqing, China. There are no specific permissions required for these sampling activities at the TongXing Waste Power Generation Plant, and the sampling activities did not involve endangered or protected species. The sampling fly ashes were dried in a thermostatic heater at 120 °C for 2 hours and sifted by a 200-mesh sieve. The elemental analysis of the MSWI fly ash samples was measured by X-ray fluorescence (XRF, 1800CCDE). The major and minor phases of the fly ash samples were measured by X-ray diffraction (Shimadzu XRD-6000) and analyzed by MDI Jade 5.0 software based on the diffraction patterns of the samples^8^. The activated charcoals (ACCs) used in the tests were manufactured by the Chemical Reagent co., LTD in Chongqing, China via the thermal decomposition method. The particle size distribution of an MSWI fly ash sample was detected and reported by a laser particle sizer (Microtrac S3500, USA). The porosity and specific surface areas of the PRB materials were measured and analyzed by a BET surface-area analyzer (ASAP 2010, Micromeritics, USA). The pH corresponding to the point of zero charge (pH_zpc_) of an ACC was determined by a zeta meter (Pen 3.0 + , Kem Inc., USA)^19^.

### Aqueous equilibrium adsorption tests

A stock solution of HMs was prepared using ZnCl_2_, PbCl_2_, CuCl_2_ and CdCl_2_ in deionized water; the amounts of these materials that were added were 250 mg/L, 25 mg/L, 100 mg/L and 15 mg/L, respectively. The additions of the HMs were determined based on the results of the leaching toxicity of the fly ash samples. Considering the high content of Ca^2+^ in the fly ash and the oxidation of aluminum wire during the EKR process, 500 mg/L of CaCl_2_ and 500 mg/L of Al_2_O_3_ were also added to the heavy metal solutions. The effect of pH on the equilibrium adsorption of the activated carbon for Zn^2+^, Pb^2+^, Cu^2+^ and Cd^2+^ was evaluated in the range of 4.0–13. Dried activated carbon in the amounts of 0.5 g, 1 g and 5 g were added to the 100-mL solution to achieve adsorption equilibrium. The reaction mixtures in the conical flasks were shaken on an oscillating shaker at 150 rpm and 25°C for 24 h. At the end of the experiment, the solutions were separated from the adsorbent by centrifugation at 10,000 rpm for 5 min. The concentrations of four HM elements in the solutions were analyzed using ICP-OES. The removal percentage of HMs from the simulated solution at equilibrium was calculated using Eq. (1):


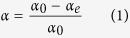


where α_0_ and α_e_ (mg/L) are the liquid-phase concentrations of HMs at the initial and equilibrium states, respectively. All experiments were conducted in triplicate, and the average values were used in the data analysis below.

### EK experiments

**Figure 1:**
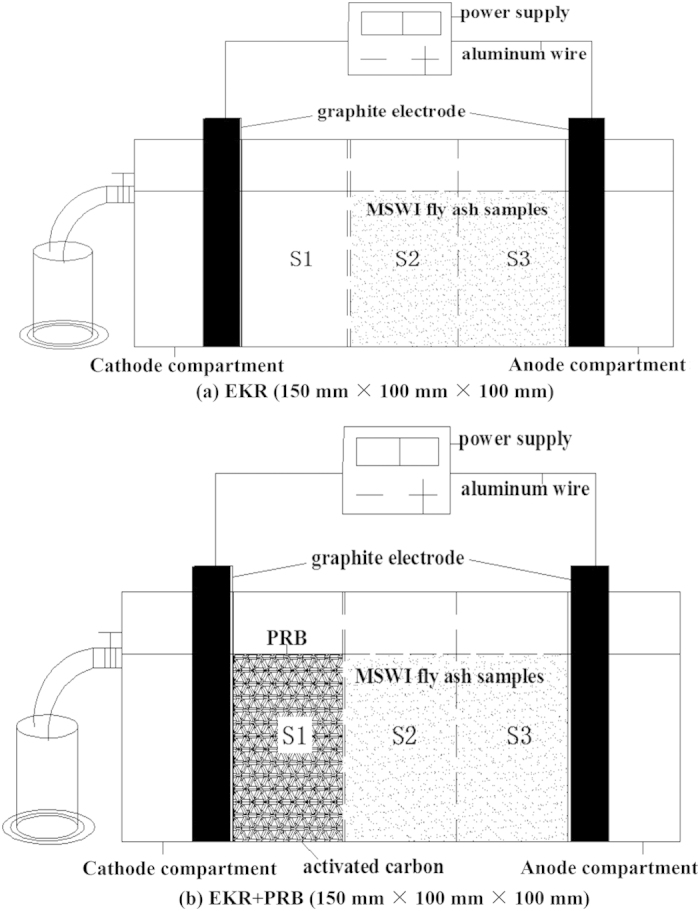
The schematic cross-section of the testing devices.

The EKR and EKR-PRB experiments were conducted in rectangular glass boxes. The schematic cross-section of the testing devices used for the corresponding experiments are shown in Fig. 1. The testing device consisted of two major parts: a chamber holding MSWI fly ash samples and the electrode compartments. The chamber (150 mm × 100 mm × 100 mm) was evenly divided into three regions (S1, S2 and S3) from the cathode to anode for all experiments. S1 and S2 is separated using plastic mesh (Φ 0.25 mm). The Graphite electrodes (10 mm × 100 mm × 100 mm) were placed near the ends of the sample chamber in the electrode compartments as anodes and cathodes. For the EKR experiment, only deionized water was poured into the S1 region in the chamber before the test. For the EKR-PRB experiment, activated carbon particles were placed into the S1 region and mixed with the deionized water^9^. For the enhanced EKR-PRB (ENEKR + PRB) experiment, a given amount of oxalic acid (0.05 mol/L) was added to the PRB media region at regular intervals (e.g., every two days). The experimental conditions of the three types of batch tests are shown in Table 1. To further elevate the removal efficiencies of HMs in the fly ash and obtain optimum performance, three factors including the voltage gradient, the processing time and the oxalic acid concentration, each at three different levels, were considered to design the orthogonal tests for the enhanced EKR-PRB (ENEKR + PRB) system. The detailed information of the orthogonal tests is shown in Table 2.

### Concentration and leaching toxicity analysis

At the end of the experiments, the fly ash samples in the S2 and S3 regions were digested in a microwave to determine the total concentrations and the removal rates of the HM contaminants from the fly ash samples. The solid material (0.2 g) was mixed with 5 mL of HNO_3_, 2 mL of H_2_O_2_ (30%) and 3 mL of HF and was digested in the microwave digestion system (MDS) at temperature of 200°C for two hours (GB5085). The digestion solution was diluted to 100 mL using deionized water and then investigated by inductively coupled plasma optical emission spectrometry (ICP-OES). The leaching toxicities of Zn, Pb, Cu and Cd were determined based on the toxicity characteristic leaching procedure (TCLP)^27^. The MSWI fly ash sample (10 g) mixed with 200 mL of 0.0992 M acetic acid and 0.0643 M NaOH was shaken on a rotary shaker at approximately 300 rpm for 18 h, and the pH in the mixture was maintained at 4.93 ± 0.05. Then, the filtrate was adjusted by nitric acid and was measured by ICP-OES to determine the concentration of the HM elements. The removal rates of the HMs in a fly ash sample was calculated as Eq. (2):


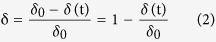


where δ is the removal rate (%) of the heavy metal in the fly ash sample, δ(t) is the HM concentration (mg/L) in the sample at time t, and 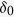 is the initial concentration (mg/L) of HMs in the sample before the experiment.

### Morphology analysis

The morphology and chemistry of the ACCs were analyzed by scanning electron microscopy with energy dispersive spectrometry (SEM-EDS). The changes of the surface structures and elemental contents on the surfaces of the PRB media before and after the tests were determined and analyzed.

### FTIR analysis

To detect the surface functional groups involved in the metal adsorption and improve the analysis of the removal mechanisms of HMs in the EKR-PRB system, the ACC samples obtained before and after the experiments were analyzed using Fourier transform infrared spectroscopy (Nicolet 5DXC FT-IR) in the range of 400–4000 cm^−1^.

### Desorption and regeneration analysis

The loaded ACC gotten from the electrolyzer were added to 100 mL of 0.05 mol/L NaNO_3_ solution being stirred for 4–12 h. The unloaded PRB media were separated from the solution and washed with deionized water three times to remove the ions and inorganic acid adsorbed on the surface. The filtrate was doped and concealed in sewage collection system. The treated solid media were dried at temperature of 100°C to be recycled for the next EKR-PRB process. The methods of MDS and ICP-OES were used in combination to detect and calculate the desorption efficiencies of HMs in ACC^25^.

## Results and Discussion

### Elemental properties of the fly ash and PRB media

**Figure 2:**
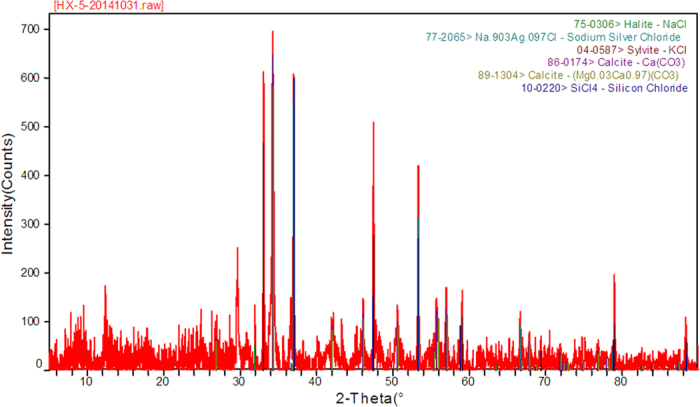
X-ray diffraction pattern of MSWI FA sample.

**Figure 3:**
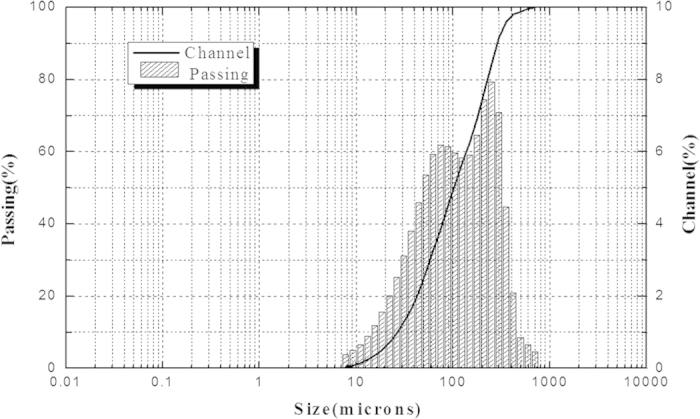
The result of size distribution of fly ash.

The semi-quantitative results of the elemental and major phase analyses of the fly ash samples are shown in Table 3 and Fig. 2, respectively. The major elements in the MSWI fly ash samples are Ca, O and Cl, and the cumulative percentage of these three elements is 82.9269%. There are four measurable HM elements (Zn, Pb, Cu and Cd) that were detected in the samples, and their cumulative percentage is 0.9666%. The total concentration of these four HM elements in the original fly ash sample measured by ICP-OES from the digestion solution are 5256.25 mg/kg, 2047.26 mg/kg, 593.33 mg/kg and 149.02 mg/kg, respectively, and their total concentration is 8045.86 mg/kg (Table 4). The major phases contained in the fly ash samples include halite (NaCl), sodium sliver chloride (Na_0.903_Ag_0.097_Cl), sylvite (KCl), calcite (CaCO_3_, Mg_0.03_Ca_0.97_CO_3_) and silicon chloride (SiCl_4_). By standardizing the fom value (<10) as the matching criterion in the MDI Jade software, the HM elements in the fly ash sample are found to exist primarily in the form of minor and trace phases. The detailed information of the phases of HMs is shown in Table 4. The size distribution of the fly ash samples is shown in Fig. 3.

The BET surface area, total pore volume and average pore diameter of the ACC are 852.83 m^2^/g, 0.501 cm3/g and 4.41 nm, respectively. The average pore diameter of 4.41 nm indicates that the PRB material is in the mesoporous region (R = 2–50 nm). The pH_zpc_ of the PRB media is 2.6, which indicates that when the pH of the solution in the electrolyzer is greater than that value, the particle surface of the adsorbent will be negatively charged, and vice versa.

### Equilibrium adsorption of HMs with the PRB material in the aqueous phase

Batch equilibrium tests were conducted to study the equilibrium adsorption of the HMs on the selected ACC media. The effects of the initial pH and the adsorbent dosage on the adsorption uptake or the removal efficiency were investigated in this study; other factors, such as the contact time and shaking speed were held constant. In the EKR process, the processing time of the fly ash usually lasted two to four weeks, which was significantly longer than the contact time of the equilibrium adsorption tests, which determined the processing time for the coupled system.

### pH effect

**Figure 4:**
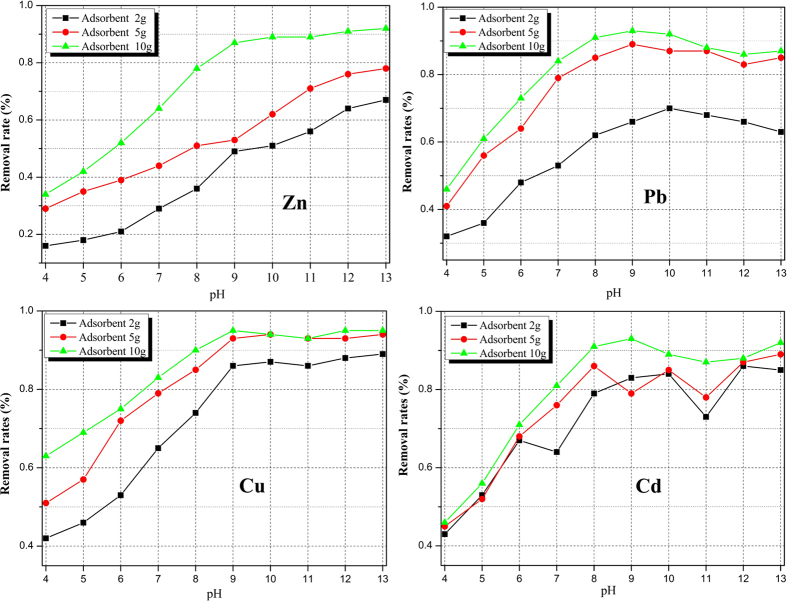
Effects of pH of the solution and adsorbent dosage on Zn, Pb, Cu and Cd removal by activated carbon.

The removal efficiency of the HMs from the simulated solution is highly controlled by the pH of the liquid phase, which can significantly affect the electrical properties of the adsorbent, the ionization degree and the speciation of the adsorbate. The removal results for Zn, Pb, Cu and Cd after the equilibrium tests are shown in Fig. 4. The removal of Zn, Pb, Cu and Cd were found to be significantly higher than the counterpart in the lower pH range, which basically increased with increasing pH in the aqueous solution. The possible reasons for this were concluded to be the reduction of competing ions, such as hydrogen ions, Al^3+^ and Ca^2+^; the formation of monovalent cations (e.g., Me(OH)^+^) or non-soluble hydroxides (e.g., Me(OH)_2_); and the combination of HM ions with the functional groups on the surface of the adsorbent. In this study, a large amount of Al^3+^ and Ca^2+^ ions were added to the solution to accurately simulate the adsorption environment of the electrolyte; the coprecipitation between the hydroxides of Al^3+^, Ca^2+^ and HMs should also be considered in addition to the adsorption competition. As the pH continued to increase, some anions of Pb and Cd, such as [Pb(OH)_3_]^–^, [Cd(OH)_4_]^2–^ and [Cd(OH)_3_]^–^, were produced in the solution, repelling the adsorption of the negatively charged surface of the adsorbent, which was verified to some extent by the down slope and fluctuation of the removal results for Pb and Cd (Fig. 4).

### Adsorbent dosage effect

Adsorption of Zn, Pb, Cu and Cd at different adsorbent doses (2, 5 and 10 g/100 mL) was conducted at a constant concentration of HMs in the solution to analyze the effect of the dosage on the removal efficiency of the HM ions. The amount of the adsorbent dosage was found to be associated with the pollutant retention, adsorption and desorption efficiencies. The removal results of the HMs in the equilibrium tests (Fig. 4) showed that the percentage of adsorption of the HMs all increased with an increased adsorbent dose. In either pH condition for either heavy metal ion, the adsorption of the 10 g/100 ml case was always the highest; this can be explained by the fact that as the adsorbent dosage increased, more surface area and thus more active sites were available for binding or adsorption of the HM ions.

## The EKR-PRB coupled system

**Figure 5:**
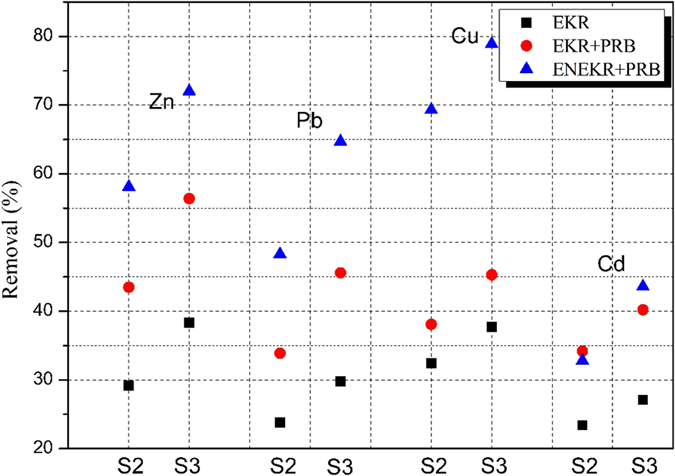
Removal rates (δ) of HMs from the fly ashes in the S2 and S3 regions of the sample chamber.

Three types of batch EKR-PRB tests (Table 1) were conducted to measure the effect of the coupled system on the removal of HMs from MSWI fly ashes based on the results of the aqueous equilibrium adsorption tests. The removal rates (δ) of HMs from the fly ashes in the S2 and S3 regions of the sample chamber are shown in Fig. 5. The corresponding analysis of statistical significance between different experimental systems by using function ttest2 (Matlab) is listed in Table 5. It is clear that the difference on the removal of HMs among systems of EKR, EKR + PRB, ENEKR + PRB) is significant. As shown in Fig. 5, the δ values of the HMs (Zn, Pb, Cu and Cd) in the coupled system were significantly elevated in the S2 or S3 region compared to those under the EKR process. In the coupled process, the δ values of the HMs in the ENEKR + PRB (oxalic acid addition) process in the S3 region (i.e., near the anode) were all higher than that in the EKR-PRB test. Based on the coupled system, the addition of oxalic acid is shown to have some effect on the removal of the HMs, particularly of Cu. The underlying reasons for this phenomenon might be the increase of the pollutant desorption from the surface of the fly ash matrix and the enhancement of the complex reaction on the surface of the activated carbon granules.

### pH changing

**Figure 6:**
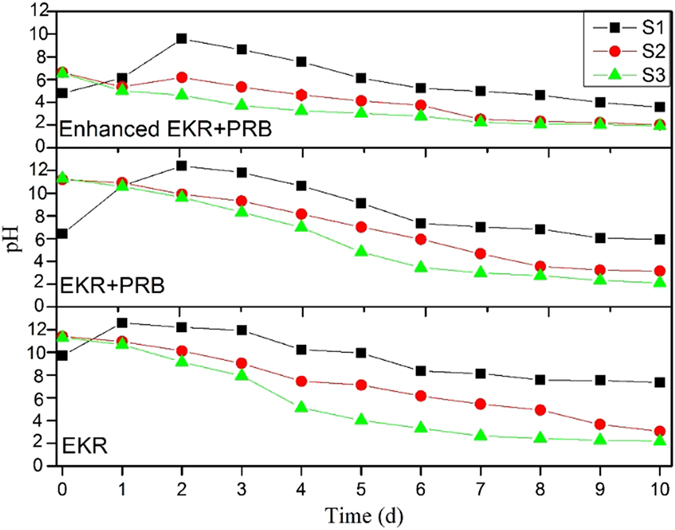
pH changing trends of the batch EKR-PRB tests.

The migration of HM ions in the porous water of the fly ash sample was controlled by several interacting mechanisms during the EKR process, including the desorption and adsorption of contaminants, advection, diffusion and the migration of the acid front to the direction of the cathode electrode. However, the chemical phases and speciation of the mineral phases, the conditions of the electroosmosis and the polarization of the fly ash matrix and the electrodes were all significantly affected by the pH distribution in the sample chamber. The electrolysis of water occurs in the electrode reservoirs, generating hydrogen ions (H^+^) at the anode and hydroxyl ions (OH^–^) at the cathode with the production of hydrogen (H_2_) and oxygen (O_2_). Under an electrical field in the chamber, the mobility of the H^+^ ions was nearly twice as larger as that of the OH^−^ ions, which would dominate the EKR system in the middle and late phases of the experiment. The H^+^ ions tended to exchange with the cationic contaminants on the matrix surface, causing the release of the corresponding contaminants. The trends of the pH of the batch EKR-PRB tests in the regions of the sample chamber are shown in Fig. 6. The corresponding analysis of statistical significance on pH variations among three experimental systems is displayed in Table 5. The difference between ERK and EKR + PRB is insignificant which means the placement of PRB in the electrolyzer does not significantly influence the pH distribution during the experiment. The significance of EKR-ENEKR + PRB and EKR + PRB-ENEKR + PRB reflects the obvious effect of oxalic acid on the pH variations. The general trends of the pH in all three conditions (Fig. 6) showed a similar pattern: the pH values near the anode in all cases gradually decreased with time, and those near the cathode first increased and then decreased. The pH values in the S2 region were effectively constrained between those in the S1 and S3 regions after the first day of the test. A pH gradient that existed between the S2 and S3 regions was higher than that between the S1 and S2 regions. The possible reasons for these findings were the transformation of the interface (i.e., from solid-solid to solid-liquid) and the clogging of the pores caused by the production of the chemical precipitation. As shown in Fig. 5, the addition of the oxalic acid in the ENEKR + PRB process can mitigate the observed pH gradient and narrow the difference between the S2 and S3 regions’ pH values to some extent.

### Current density changing

**Figure 7:**
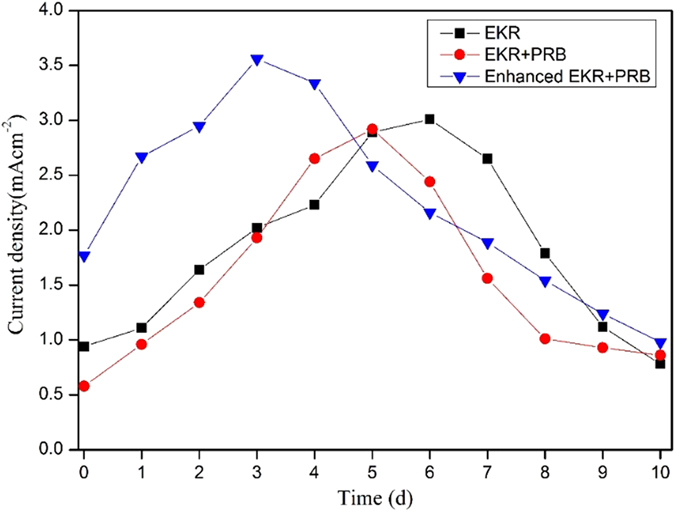
Current density changing trends of the batch EKR-PRB tests.

The trend of the current density in the batch coupled tests is shown in Fig. 7. The current density was calculated by the measured total current through the cross section of the sample chamber in the electrolyzer. The addition of PRB and oxalic acid cannot significantly affect the current density changing (Table 5). The initial current density in the EKR and EKR + PRB tests was 0.5–1.0 mAcm^−2^, as shown in Fig. 7. The low values at the early stages were possibly caused by the low concentration of dissolved contaminants and the slow migration of the acid front. As the pH changed, the general trends of the current density under the three conditions also showed a similar pattern: the current density first increased and then decreased. The fastest changes appeared in the early and middle phases of the experiments. The initial increase in the current density was primarily caused by the elevated concentration of the dissolved free ions, the fluent migration of the acid front and the continuing release of the metal ions. From the variations of the current density shown in Fig. 7, it is clear that the use of the PRB (i.e., activated carbon) and the addition of the complex reagent (i.e., oxalic acid) caused the corresponding peaks shift to the left compared to the trend of the EKR process, which showed that the time of the maximum current density in the electrolyzer appeared earlier under the coupled system. At the end of the experiment, the current density under all conditions gradually decreased to a low state. The decrease of the current density was primarily due to the concentration polarization, the increase of the resistance caused by the decline of the ion concentration in the porous water and the production of some precipitates.

### Removal optimization of HMs in the enhanced EKR-PRB coupled system

**Figure 8:**
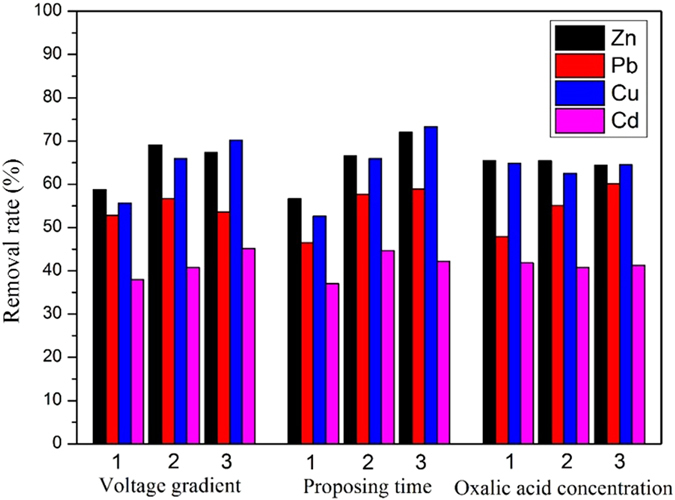
Mean removal rates of HMs under the condition of three experimental factors with three levels.

To further increase the removal rates of the HMs (Zn, Pb, Cu and Cd) from the MSWI fly ash samples and determine the most significant factor, orthogonal tests were designed based on the ENEKR + PRB batch tests. The removal results of the corresponding HMs in the S3 region under the designed experimental condition (L9(34)) are shown in Table 2. As shown, the maximum removal of Zn was 78.34%, which was obtained at a 2-V/cm voltage gradient, a 15 d of processing time and 0.1 mol/L of oxalic acid; this was 1.045, 0.389 and 0.088 times higher than that under the conditions of the EKR only, EKR + PRB and ENEKR + PRB in the above batch coupled tests (Fig. 5), respectively. The maximum removal of Pb was 69.34%, which was achieved under the condition of a 1.5-V/cm voltage gradient, 10 d of processing time and 0.2 mol/L of oxalic acid; this was 1.327, 0.521 and 0.072 times higher than those under the corresponding conditions in the above batch coupled tests, respectively. The maximum removal rate for Cu was 84.14% when a 2-V/cm voltage gradient, 15 d of processing time and 0.1 mol/L were used; this was 1.126, 0.769 and 0.0157 times higher than that under the corresponding conditions of the batch coupled tests. The maximum removal rate of Cd was 49.23% when a 2-V/cm voltage gradient, 10 d of processing time and 0.05 mol/L of oxalic acid were used; this was 0.817, 0.2246 and 0.1291 times greater than that under the corresponding conditions of the batch coupled tests, respectively. To compare the significances of each experimental factor (i.e., voltage gradient, proposing time and oxalic acid) at each level, the mean removal rates of the HMs with the three experimental factors at three different levels were calculated, and the results are shown in Fig. 8; the results of the variance analysis of the orthogonal tests are also shown in Table 6. From the analytical results (Table 6, Fig. 8), it was clear that the variations of the voltage gradient and the processing time have significant effects on the removal of Cu and Cd in the coupled system. Additionally, changes in the amount of oxalic acid added yielded more influence on the removal of Pb than on the other HMs; however, this was not significant (0.238 > 0.05). In general, the proposing time is more significant than the other two factors on the removal rates of the HMs in the coupled system for the MSWI fly ash samples.

## Removal mechanisms of HMs in the EKR-PRB system

### Changes of phases

**Figure 9:**
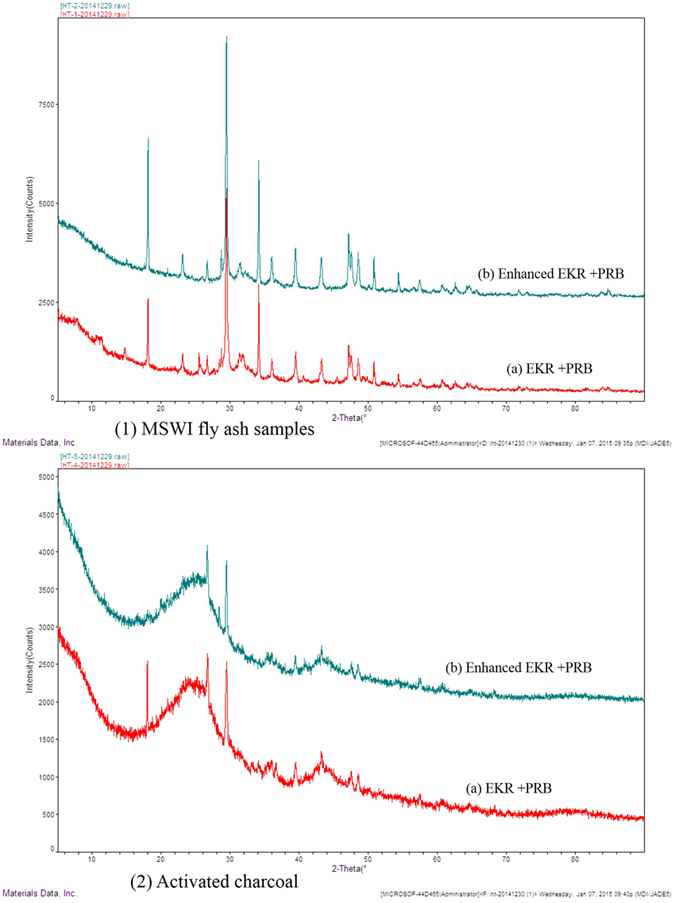
The XRD patterns of MSWI fly ash samples near the interface and PRB media in S1 region after the experiments.

As shown in Fig. 6, there were always pH gradients between the solid-liquid interface (i.e., between S2 and S3) through the coupled experiments, which was usually considered to be one of the primary reasons for the increasing mobility resistance of the ions, the concentration polarization of the electrolyte and the transformation of specific phases. Thus, to clearly understand the redistribution of HMs in the sample region during the tests and analyze the possible adsorption mechanisms of the PRB media (i.e., ACC), it is necessary to detect the changes in the phases of the MSWI fly ash samples and the PRB media near the interface in the ERK-PRB and ENEKR + PRB systems after the remediation experiments. The XRD patterns of the MSWI fly ash samples near the interface and the PRB media in the S1 region after the coupled experiments are thus shown in Fig. 9, and the corresponding phase results of the MSWI fly ash samples and the ACC are shown in Table 7. The experiments were conducted at a voltage gradient of 2 V/cm with 15 d of disposing time and with 0.1 mol/L of oxalic acid in the ENEKR + PRB experiment. From Fig. 9, the primary peaks at different 2θ values are generally found to be similar regardless of the MSWI fly ash or PRB media used in the two different systems. As shown in Table 7, the MSWI fly ash in the ENEKR + PRB system had fewer phase species than the phase results of the MSWI fly ash in the EKR + PRB system, which indicated that more contaminants were dissolved, and the migration of these ions was more efficient in the sample regions. Based on these findings, more phase species that were adsorbed in the ACC in the oxalic acid electrolyte were detected than those in the EKR + PRB system, which was directly reflected the increasing adsorption capacity of the ACC in the ENEKR + PRB system.

### Changes of morphologies

**Figure 10:**
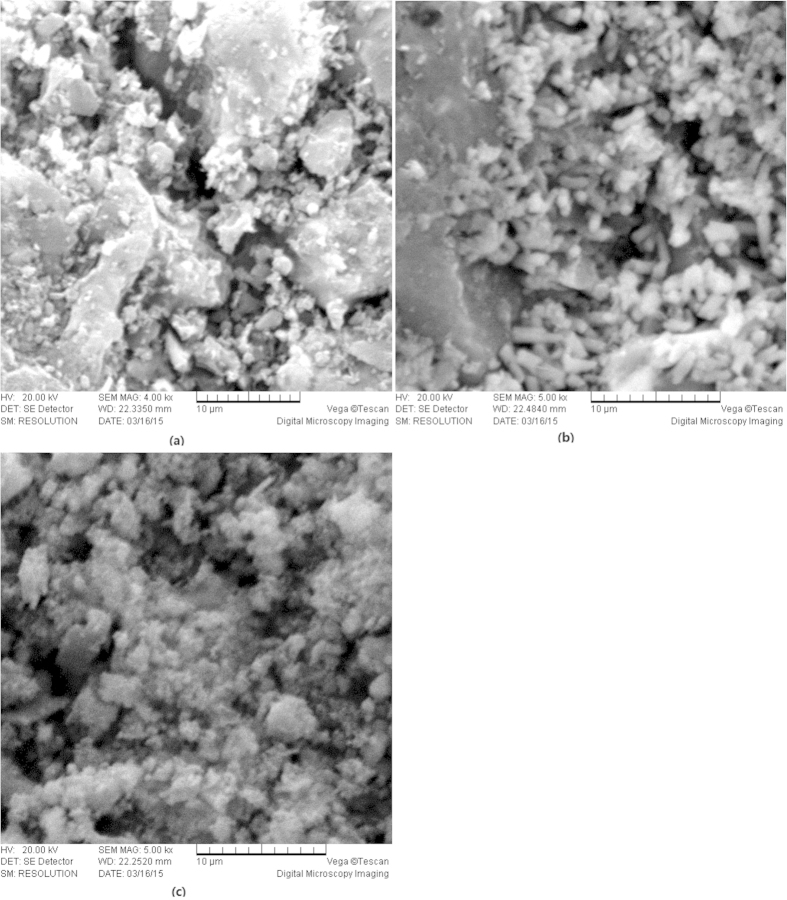
Scanning electron micrographs. (**a**) raw activated charcoal (×4000), (**b**) activated charcoal from EKR + PRB system (×5000), (**c**) activated charcoal from enhanced EKR + PRB system (×5000).

**Figure 11:**
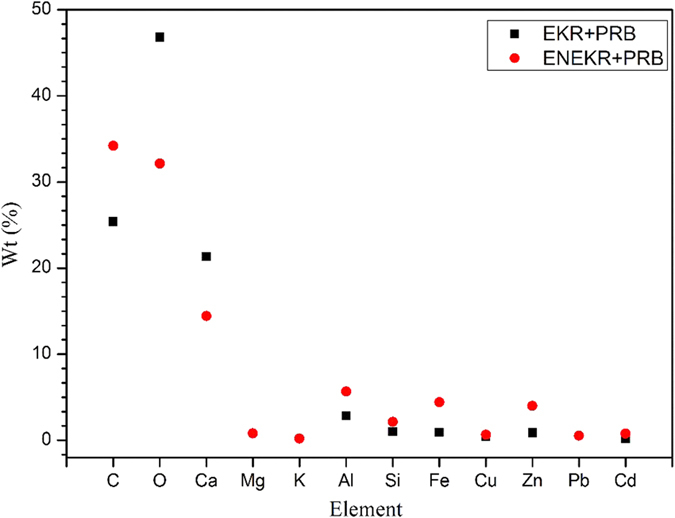
The quantitative elemental composition of composite according to the EDS spectra of activated charcoal from EKR + PRB and ENEKR + PRB system.

After the remediation experiments, the samples of the unused raw ACC and the used ACC material from the EKR + PRB and ENEKR + PRB systems were scanned by SEM-EDS. The scanning electron micrographs and the corresponding quantitative elemental composition from the EDS spectra are shown in Figs 10 and 11, respectively. Based on the figures, the surface of the ACC from the EKR + PRB system (Fig. 10b) was filled with many granular sediment particles (i.e., particle precipitation) compared with the original ACC (Fig. 10a); also, the flocculated coagulation on the media surface from the ENEKR + PRB process was clearly observed (Fig. 10c). Relative to the weight percentage (Wt%) of the elemental composition (Fig. 11) from the EDS spectra, the Wt values of carbon (C), oxygen (O) and calcium (Ca) on the surface of the ACC from the ENEKR + PRB system were significantly lower than the corresponding parts from the non-enhanced test, whereas those of the other elements, particularly including the HMs (Zn, Pb, Cu and Cd), were all higher. The two different morphologies indicated the different adsorption processes in the two systems, and the quantitative elemental composition directly illustrated the effectiveness of the doping oxalic acid to enhance the HM adsorption on the surface of the ACC during the process of the coupled experiments.

### FTIR analysis

**Figure 12:**
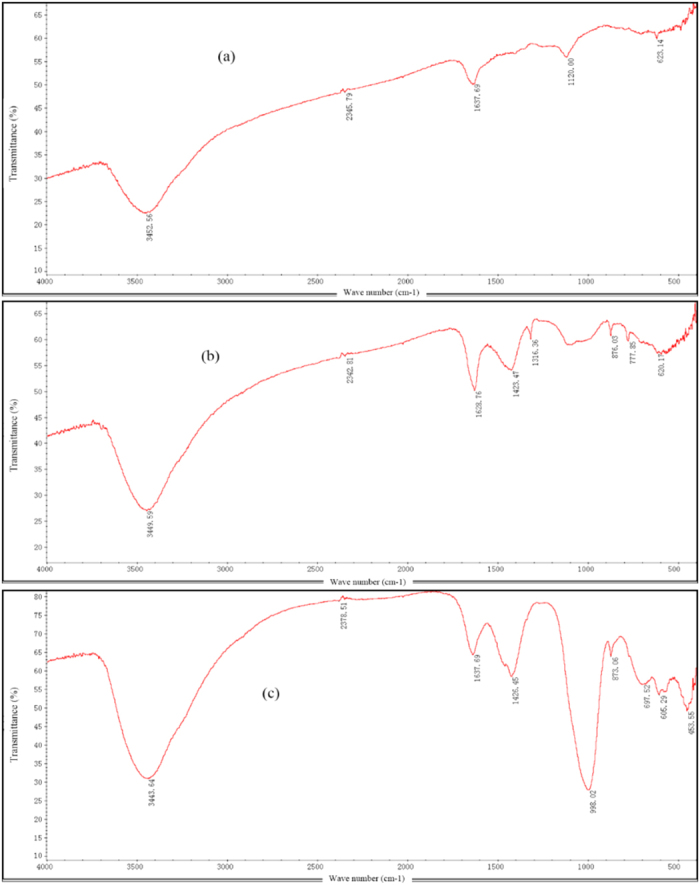
FTIR spectra of (**a**) raw activated charcoal, (**b**) activated charcoal from EKR + PRB system, (**c**) activated charcoal from enhanced EKR + PRB system.

Fourier infrared spectroscopy is usually considered to be an effective method to qualitatively or semi-quantitatively determine the chemical elements present and analyze the structural characteristics and functional groups of organic, inorganic and compound molecules. Through the comparison and the analysis of the characteristic absorption peaks in the spectrogram, the change of the functional groups on the surface of the adsorbent can be clearly determined. The FTIR spectra of the raw activated coal (a) and ones from the EKR + PRB (b) and ENEKR + PRB (c) systems have been investigated and are shown in Fig. 12. The FTIR spectra of the raw ACC (Fig. 12(a)) shows spectrum bands of –OH stretching at 3452.56 cm−1, C = C shift stretching at 1637.69 cm−1, C-O-C bending at 1120.00 cm−1, and C-H deformation at 623.14. The spectrum bands of the ACC sample from the EKR + PRB system include -OH stretching at 3449.59 cm−1, C = C stretching at 1628.76 cm−1, –OH in-plane at 1423.47 cm−1, C = N stretching at 1316.36 cm−1, C-O-C bending at 1316.00 cm−1, and C-H bending at bands of 777.85 cm−1 and 620.17 cm−1. The spectrum bands of –OH stretching at 3443.64 cm−1, C = C stretching at 1637.69 cm−1, C-H deformation at 1426.45, C-O-C bending at 998.02 cm−1, 873.06 cm−1 and 679.52 cm−1, C-H bending at 605.29 cm−1 are also present. The surface chemistry of the raw ACC and the ACC samples from the two different coupled systems were found to be different: some functional groups were shifted, disappeared or added due to the different chemical pathways present, including the changing pH environment in the electrolyte, organic acid grafting, adsorption and desorption of the HMs and protons between the adsorbate and the adsorbent. Compared the spectrograms of ACC samples from the systems of EKR + PRB and ENEKR + PRB with the raw one, the appearance of –OH in-plane and C = N stretching, and the increase of C-O-C bending spectrum bands directly reflect the influence of hydroxyl (OH^-^) produced by hydrolysis on the reactive sites, and the oxalic grafting and the adsorption of some inorganic pollutant on the ACC grains.

### Leaching toxicity comparison

The leaching toxicity characteristics used to simulate the leaching of HMs through a landfill are also considered to be an indicator that reflects the final remediation efficiency for contaminated solid. The leaching toxicities of the fly ash found in the S2 and S3 regions of the sample chamber after the experiment are shown in Table 8. In the raw MSWI fly ash, the leaching toxicities of Zn, Pb, Cu and Cd were 145.21 mg/L, 8.79 mg/L, 21.34 mg/L and 5.73 mg/L, respectively. The leaching activity of Zn was found to be higher than that of the other three elements under the same testing conditions. Comparing the leaching results of the fly ash sample from the different processing system (EKR, EKR + PRB, ENEKR + PRB) with each other, the leaching value of each HM from the ENEKR + PRB system was lowest and showed the least difference in their values between the S2 and S3 regions. Additionally, although the leaching toxicity of the sample from the EKR + PRB test was lower than that from the enhanced coupled test (ENEKR + PRB), relative to the uncoupled treat (EKR), the leaching toxicity of each HM was significantly decreased, which proves the effectiveness of loading the activated charcoal PRB into the electrolytic cell during the electroremediation process.

### HMs desorption and regeneration of PRB media

**Figure 13:**
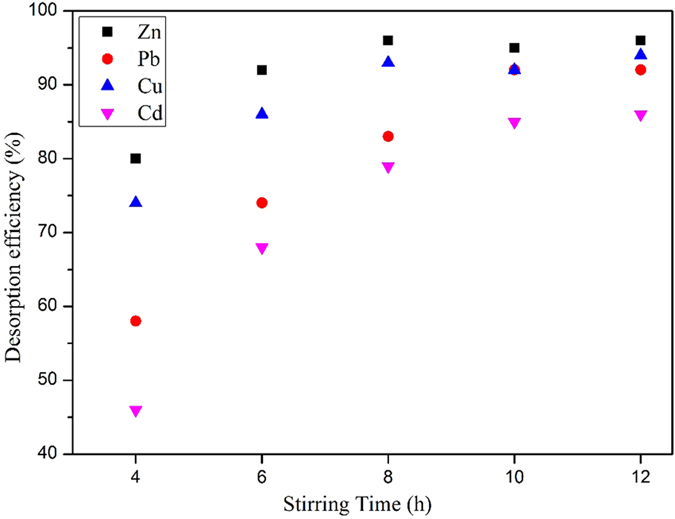
Desorption efficiencies of HMs from the loaded ACC.

HMs desorption experiments were carried out to transfer HMs into the inorganic acid solution and regenerate the PRB media. The regenerated ACC will be available for recycling use in the following experiments. As shown in Fig. 13, at 8h of stirring time, the desorption of Zn and Cu have been stable with efficiencies of 96% and 93% respectively, and at 10h, all the four HMs (Zn, Pb, Cu and Cd) have entered into plateau period with efficiencies of 95%, 92%, 92% and 85%, and only small variation can been recorded at 12h. The bubbles generated by the stirring plates in the middle of a breaker intensely shear the ACC granules facilitating cation-exchange happened on the surface of pores.

## Conclusions

Regarding the toxicity of the MSWI fly ash specimens, four measurable and detectable HM elements, Zn, Pb, Cu and Cd, were found in the original fly ash sample and had a cumulative percentage of 0.9666% in the sample and corresponding concentrations of 5256.25 mg/kg, 2047.26 mg/kg, 593.33 mg/kg and 149.02 mg/kg, respectively. Compared to those of the EKR process, the δ values of the HMs in the coupled systems (EKR + PRB, ENEKR + PRB) in the S2 and S3 regions were all higher, and that in the S3 region in the ENEKR + PRB system reached the maximum value for each HM. These results indicated that the proposed combined method could improve the remediation efficiency, and the addition of oxalic acid to the PRB media before the coupled system could further enhance the remediation process by reducing the pH gradient between the S2 and S3 regions and shifting the current density peaks to the left of the x axis. Based on the results of the designed optimization tests, the maximum removals of Zn, Pb, Cu and Cd were achieved under different experimental conditions. Based on the analysis of each experimental factor, the voltage gradient and processing time were found to have significant effects on the removal of Cu and Cd, and the addition of oxalic acid was found to have more influence on the removal of Pb than on those of the other HMs. Generally, the processing time has a more significant effect on the removal rates of the HMs in the enhanced coupled system for the MSWI fly ash samples than the other two factors. In the discussion of the removal mechanism of the HMs from the MSWI fly ash, the specimen from the ENEKR + PRB system was found to have significantly fewer phase species than those from the EKR + PRB system, which indicated that more contaminants were dissolved, and the migration of the ions was more efficient in the electrolyzer. However, additional phase species that adsorbed in the ACC in the oxalic acid electrolyte were detected compared to that in the EKR + PRB system, which demonstrated the boosting adsorption capacity of ACC in the ENEKR + PRB system. Two different morphologies on the surfaces of the ACC from the EKR + PRB and ENEKR + PRB systems showed that different adsorption processes were occurring during the tests in the two systems. The corresponding quantitative elemental compositions directly illustrated the effectiveness of the doping oxalic acid to enhance the HM adsorption on the PRB media. The specimen remediated by the ENEKR + PRB system showed the lowest leaching value for each HM in the S2 and S3 regions.

## Additional Information

**How to cite this article**: Huang, T. *et al.* Heavy metal removal from MSWI fly ash by electrokinetic remediation coupled with a permeable activated charcoal reactive barrier. *Sci. Rep.* **5**, 15412; doi: 10.1038/srep15412 (2015).

## Figures and Tables

**Table 1:** The experimental conditions of the three types of batch tests.

**Type**	**Sample Chamber**			**Electrode Electrolyte**		**Experimental Conditions**	
	**S1**	**S2**	**S3**	**Anode**	**Cathode**	VoltageGradient(V/cm)	ProposingTime (d)
EKR	Deionized water	Fly ash	Fly ash	Deionized water	Deionized water	1.5	10d
EKR + PRB	Activated carbon	Fly ash	Fly ash	Deionized water	Deionized water	1.5	10d
Enhanced EKR + PRB	Activated carbon + oxalic acid	Fly ash	Fly ash	oxalic acid	oxalic acid	1.5	10d

**Table 2:** The design of orthogonal tests of the enhanced EKR-PRB system (Three factors with three levels, L_9_(3^4^)).

TestNo	**Voltage Gradient(V/cm)**	ProposingTime(d)	Oxalic Acidconcentration(mol/L)	**Removal rate (%)**			
				**Zn**	**Pb**	**Cu**	**Cd**
T-1	1 (1 V/cm)	1 (5d)	1 (0.05 mol/L)	54.32	42.12	47.16	34.91
T-2	2 (1.5 V/cm)	1	2 (0.1 mol/L)	57.11	46.33	53.26	36.15
T-3	3 (2 V/cm)	1	3 (0.2 mol/L)	58.49	51.12	57.45	40.02
T-4	1	2 (10d)	2	60.78	56.45	54.12	39.87
T-5	2	2	3	73.65	69.34	70.43	44.79
T-6	3	2	1	65.25	47.32	73.12	49.23
T-7	1	3 (15d)	3	61.11	59.83	65.67	39.12
T-8	2	3	1	76.65	54.34	74.23	41.34
T-9	3	3	2	78.34	62.45	80.14	46.25

**Table 3:** Semi-Quantitative results of the element analysis.

**Element**	**E-Content (%)**	**Oxide**	**O-Content (%)**
Ca	34.8684	CaO	53.6008
O	32.4644	—	—
Cl	15.5941	—	—
Na	4.5340	Na_2_O	6.3662
K	3.7446	K_2_O	4.9066
S	2.6689	SO_3_	7.0578
Si	1.8508	SiO_2_	4.1694
Mg	1.0573	MgO	1.8378
Fe	0.8769	Fe_2_O_3_	1.4133
Zn	0.6364	ZnO	0.8923
Al	0.5204	A1_2_O_3_	1.0331
P	0.3279	P_2_O_5_	0.7939
Ti	0.2289	TiO_2_	0.4284
Pb	0.2070	PbO	0.2518
Br	0.1006	—	—
Cu	0.0691	—	—
Cd	0.0541	—	—
Mn	0.0441	MnO	0.0639

**Table 4:** The phases and the total concentration of HMs in the original MSWI fly ash sample.

**Element**	**Phase**	ChemicalFormula	TotalConcentration(mg/kg)
Zn	Zincite	ZnO	5256.25
	Zinc Silicate	ZnSiO_3_	
Pb	Lead Oxide	PbO	2047.26
	Lead Chloride-HP	PbCl_2_	
Cd	Cadmium Oxide	CdO	149.02
	Cadmium Chloride	CdCl_2_	
Cu	Copper Chloride	CuCl_2_	593.33

**Table 5:** Statistical significance between different experimental systems.

**ttest2**		**h**	**Sig.**^a^	**Df **b	**Std**^c^
Removal rate	EKR-EKR + PRB	1	0.0027	14	6.5621
	EKR-ENEKR + PRB	1	2.9940e-004	14	11.8468
	EKR + PRB-ENEKR + PRB	1	0.0189	14	12.3009
pH value	EKR-EKR + PRB	0	0.7231	64	3.2029
	EKR-ENEKR + PRB	1	2.8877e-005	64	2.6909
	EKR + PRB-ENEKR + PRB	1	9.1453e-005	64	2.6295
Current density	EKR-EKR + PRB	0	0.4342	20	0.8014
	EKR-ENEKR + PRB	0	0.2561	20	0.8225
	EKR + PRB-ENEKR + PRB	0	0.0673	20	0.8276

Default significance.

^a^0.05.

^b^degrees of freedom.

^c^standard variance.

**Table 6:** The results of variance analysis of the orthogonal tests.

**Sources**	**Element**	**Type II Sum of Squares**	**df**	MeanSquare	**F**	**Sig.**
Voltage gradient	Zn	185.678	2	92.839	2.130	0.320
	Pb	24.907	2	12.454	0.354	0.738
	Cu	337.518	2	168.759	14.218	0.066
	Cd	79.061	2	39.531	20.287	0.047
Proposing time	Zn	365.318	2	182.659	4.190	0.193
	Pb	278.884	2	139.442	3.969	0.201
	Cu	661.063	2	330.531	27.847	0.035
	Cd	90.683	2	45.341	23.269	0.041
Oxalic Acid concentration	Zn	1.967	2	.983	0.023	0.978
	Pb	224.432	2	112.216	3.194	0.238
	Cu	9.571	2	4.786	0.403	0.713
	Cd	1.718	2	0.859	0.441	0.694
Error	Zn	87.180	2	43.590		
	Pb	70.272	2	35.136		
	Cu	23.739	2	11.869		
	Cd	3.897	2	1.949		

Computed using alpha = 0.05.

**Table 7:** The phase results of MSWI fly ash samples near the interface and PRB media in S1 region after the coupled experiments.

Phasetype	**MSWI fly ash**		**Activated charcoal**	
	**(a) Phase**	**(b) Phase**	**(a) Phase**	**(b) Phase**
Major	Ca(OH)2 Ca(CO3)	Ca(OH)2 Ca(CO3)	CaCO3 SiO2 NaAlH4	CaCO3 SiO2 Mg5H2W12O42(H2O)3 (C6H9NO3)n
Minor	(Mg0.03Ca0.97)(CO3) (Mg0.064Ca0.936)(CO3) Ca(SO4)(H2O)0.662 Ca(SO4)(H2O)0.67	(Mg0.03Ca0.97)(CO3)	(Mg0.06Ca0.94)(CO3) Cu2O (NH2)2SO2	Ba2Cu(PO3)8 C17H251NO2·H2O C45H33AlO6 C16H40Cl4N2Zn Mn(HSO4)2(H2O) (Zn,Mg,Mn)4Zn3(CO3)2(OH)10
Trace	C16H40Br4N2Zn Pb4(NO3)2O3	—	Ba(Bi0.4Pb0.6)O3	Ba(PbO3)

(a) EKR + PRB (b) Enhanced EKR + PRB.

**Table 8:** Leaching toxicity of the fly ashes in the S2 and S3 regions of the sample chamber after the experiment.

**HM**	**Region**	**Leaching toxicity (mg/L)**			
		**EKR**	**ERK** **+** **PRB**	**ENEKR** **+** **PRB**	**Raw**
Zn	S2	83.2	60.73	32.24	145.21
	S3	110.2	76.23	34.11	
Pb	S2	1.12	0.61	0.14	8.79
	S3	2.02	0.72	0.23	
Cu	S2	7.12	5.12	3.14	21.34
	S3	9.67	5.67	3.32	
Cd	S2	1.21	0.87	0.1	5.73
	S3	2.01	0.92	0.12	
